# Hydrogen sulphide reduces hyperhomocysteinaemia‐induced endothelial ER stress by sulfhydrating protein disulphide isomerase to attenuate atherosclerosis

**DOI:** 10.1111/jcmm.16423

**Published:** 2021-03-06

**Authors:** Shan Jiang, Wenjing Xu, Zhenzhen Chen, Changting Cui, Xiaofang Fan, Jun Cai, Yongsheng Gong, Bin Geng

**Affiliations:** ^1^ Institute of Hypoxia Medicine Wenzhou Medical University Zhejiang China; ^2^ Department of Pathology Xi'an Medical University Shanxi China; ^3^ State Key Laboratory of Cardiovascular Disease Hypertension Center National Center for Cardiovascular Diseases Fuwai Hospital of Chinese Academy of Medical Sciences and Peking Union Medical College Beijing China

**Keywords:** atherosclerosis, endoplasmic reticulum stress, homocysteine, hydrogen sulphide, protein disulphide isomerase, sulfhydration

## Abstract

Hyperhomocysteinaemia (HHcy)‐impaired endothelial dysfunction including endoplasmic reticulum (ER) stress plays a crucial role in atherogenesis. Hydrogen sulphide (H_2_S), a metabolic production of Hcy and gasotransmitter, exhibits preventing cardiovascular damages induced by HHcy by reducing ER stress, but the underlying mechanism is unclear. Here, we made an atherosclerosis with HHcy mice model by ApoE knockout mice and feeding Pagien diet and drinking L‐methionine water. H_2_S donors NaHS and GYY4137 treatment lowered plaque area and ER stress in this model. Protein disulphide isomerase (PDI), a modulation protein folding key enzyme, was up‐regulated in plaque and reduced by H_2_S treatment. In cultured human aortic endothelial cells, Hcy dose and time dependently elevated PDI expression, but inhibited its activity, and which were rescued by H_2_S. H_2_S and its endogenous generation key enzyme‐cystathionine γ lyase induced a new post‐translational modification‐sulfhydration of PDI. Sulfhydrated PDI enhanced its activity, and two cysteine‐terminal CXXC domain of PDI was identified by site mutation. HHcy lowered PDI sulfhydration association ER stress, and H_2_S rescued it but this effect was blocked by cysteine site mutation. Conclusively, we demonstrated that H_2_S sulfhydrated PDI and enhanced its activity, reducing HHcy‐induced endothelial ER stress to attenuate atherosclerosis development.

## 1 INTRODUCTION

Hyperhomocysteinaemia (HHcy), defined by an elevated serum total homocysteine (Hcy) level (>15 μM), is an independent risk factor for atherosclerosis.^1^ Elevated Hcy induced endothelial dysfunction with impairing endothelial‐dependent dilation, increasing endothelial inflammation and pro‐thrombotic condition, and promoting endoplasmic reticulum stress (ER stress).^2^ ER stress is an intracellular adaptive response, characterized by molecular chaperone (such as glucose‐regulated proteins 78, GRP78) expression increasing and unfolded protein aggregation. Homocysteine (Hcy) induced persistent ER stress with unfolded protein response (UPR), causing caspase‐12–dependent endothelial apoptosis.^3^ The endothelial dysfunction by HHcy exacerbated atherosclerosis development.

Hydrogen sulphide (H_2_S) is a novel gasotransmitter, dependent on cystathionine‐gamma lyase (CSE), cystathionine beta synthase (CBS) and 3 mercaptopyruvate sulphur transferase (3MST).^4^ In cardiovascular tissues, CSE is the major endogenous key enzyme for H_2_S generation.^5^ CSE/H_2_S exhibits anti‐atherogenic effects, evidenced by down‐regulating aortic CSE/H_2_S in atherosclerotic mice,^6^ global CSE knockout exacerbating ^7^ but the H_2_S donor NaHS or GYY4137 attenuating atherosclerosis development.^6^, ^8^, ^9^ More interestingly, H_2_S donor can lower serum total Hcy level by sulfhydrating CSE to increase its activity,^10^ and reduce heart and aorta oxidative stress injuries and ER stress in HHcy rats.^11^ However, the molecular mechanism of H_2_S attenuating HHcy‐induced ER stress is unclear.

Protein disulphide isomerase (PDI) is a redox‐dependent chaperone protein and key enzyme of protein folding. PDI can transfer oxidizing equivalents to an unfolding substrate, facilitating protein folding; so oxidized PDI is activated PDI.^12^ Inhibition PDI potentiated but overexpression PDI reduced ER stress and cellular toxicity by ox‐LDL in human microvascular endothelial cell.^13^ These studies also raise a question: whether H_2_S trigging PDI activity reduced HHcy‐induced endothelial ER stress, contribution to genesis of atherosclerosis. The present study aims to investigate it.

## 2 MATERIALS AND METHODS

The data that support the findings of this study are available from the corresponding author upon reasonable request.

### 2.1 Materials

DL‐homocysteine (44 925), DTT (43 815), NaHS (161 527) and GYY4137 (SML0100) were purchased from Sigma‐Aldrich (USA).

### 2.2 Atherosclerosis with HHcy mouse model

All animal care and experimental procedures complied with the Animal Management Rule of the Ministry of Health, People's Republic of China (document No. 55, 2001) and the Care and Use of Laboratory Animals published by the US National Institutes of Health (NIH Publication No. 85‐23, updated 2011). The care and use of laboratory animals were approved by the Laboratory Animal Ethics Committee of Fuwai Hospital. All of 45 male ApoE‐/‐ mice (8 weeks old, 20‐25 g, SPF grade, three mice per cage) were purchased from the Beijing Vital River Laboratory Animal Technologies Co. Ltd. The atherosclerosis with HHcy mouse model was made by drinking methionine water (1 g/kg) and feeding Paigen diet (D12109C, Research diets, USA) for 12 weeks. Randomly assigned to three treatment groups (n = 15 each): H_2_S donors NaHS (5.6 mg/kg/day) and GYY4137 (3.6 mg/kg/day) treated by intraperitoneal injection and control mice injection equal volume normal saline (0.9% NaCl), twice per day.

### 2.3 Plaque area measurement

Mice were anaesthetized by 1% sodium pentobarbital (50 mg/kg, ip); blood was collected from the angular artery and then perfused initially with 30 ml saline and then with 4% paraformaldehyde via the left ventricle. Entire aorta was fixed 10 minutes in 4% paraformaldehyde and then marinated 5 minutes in 60% isopropanol. Total plaque area was quantified by en‐face analysis of aorta. Removed adventitial fat from the aorta and stained with Oil Red O, opened longitudinally, pinned the aorta and photographed en‐face. The extent of lesion development was determined as a percentage of the total area of the aorta that was occupied by Oil Red O‐positive atherosclerotic lesions. The total plaque area in total arterial surface area was analysed by ImageJ.

8‐μm frozen aortic root slices were obtained by cutting from the apex of the heart towards the origin of the aorta (sections were mounted from the point where all three aortic valve cusps became clearly visible). The slices were stained with haematoxylin‐eosin staining. Plaque cross‐sectional area was determined by quantifying the plaque area by images ImageJ software.

### 2.4 Immunohistochemistry and immunofluorescence staining

Aortic root plaque slice (6 μm) was performed antigen retrieval, quenching endogenous peroxides and blocking (1% BSA, 1 hour), then incubated with anti‐GRP78 bip antibody (ab21685, Abcam) overnight at 4℃. After washing, the slices were incubated with HRP‐conjugated secondary antibody (ab7083, Abcam) for 1 hour at room temperature; the brown colour was developed using DAB, and then, images were acquired. Using mouse IgG replaced the primary antibody and corresponding secondary antibody as a negative control.

The slices were also incubated with primary antibody PDI (ab2792, Abcam) and CD31 (ab28364, Abcam), or with α‐SMA (EPR5368, Abcam) overnight at 4℃. Washing 3 times, then incubated with fluorescence secondary antibody anti‐mouse IgG (ab150113, Abcam) and anti‐rabbit IgG (ab150075, Abcam) in the dark box, and then, images were acquired in confocal microscope.

### 2.5 Serum lipid profile measurement

Serum lipid profile including levels of total cholesterol (TC), LDL‐cholesterol (LDL‐c) and HDL‐cholesterol (HDL‐c) was detected according to the protocol of manufacturer (Biosino Bio‐Technology, Beijing, China).

### 2.6 Plasmid transfection of PDI mutation

The human P4HB (NM_000918.4) insert pCDNA3.1 vector (pPDI) and mutation 1 (Cys53+ Cys56, M1), mutation 2 (Cys397 + Cys400, M2) and mutation 3 (Cys53+ Cys56 + Cys397 + Cys400, M3) plasmids were constructed by Shandong Vigene Biosciences.

### 2.7 Cell culture

HAEC cells were obtained from ScienCell (San Diego, CA, USA), cultured in ECM containing 10% FBS and 100 U/ml penicillin‐streptomycin. HAEC cells were planted in 6‐well plates and continuously cultured to 90% fusion, then changed the ECM without FBS, homocysteine (from 25 to 400 μM) for 24 hr or homocysteine (200 μM) for different times (from 2 to 24 h), or additional NaHS (200 μM) or GYY4137 (200 μM) were added. At the end of experiments, cells were collected for biotin switch assay, Western blot and PDI activity assay.

HEK‐293A cells were purchased from the National Infrastructure of Cell Line Resource (Beijing, China). Cells grew into 60% fusion, plasmids including pPDI, M1 to M3 were transfected by Lipofectamine 3000 (Thermo Fisher Scientific, USA), then continuously cultured for 24h, cells were collected for biotin switch assay, Western blot and PDI activity assay.

### 2.8 Western blot

Total protein from cells or tissues was lysed by RIPA buffer, and the protein content was determined by BCA Protein Assay Kit (Thermo Fisher Scientific, USA). Protein was denatured; then, 20 µg of proteins was separated by SDS‐PAGE gels and transferred into nitrocellulose membranes (1 620 113, Bio‐Rad, USA). After blocking with 5% milk in TBST (50 mM Tris‐HCl, 150 mM NaCl and 0.1% Tween 20) for 1 h, membranes were incubated with primary antibodies against PDI, GRP78, XBP1s (24868‐1‐AP, ProteinTech, China), cleaved‐caspase 12 (#2224, Cell Signaling Technology, USA) (1:1000) or GAPDH (#97166, Cell Signaling Technology, 1:5000) overnight at 4 ℃. Washed with TBST for 3 times (5 min per time), then incubated with an HRP‐conjugated secondary antibody (1:10 000, ab7061 or ab6802, Abcam) for 1 hr at room temperature. After washing, images of chemiluminescence using ECL (Thermo Fisher) were acquired. Grayscale semi‐quantitative analysed by ImageJ.

### 2.9 Biotin switch assay for protein sulfhydration

The assay was performed as described by Mustafa et al.^14^ Cells were homogenized in HEN buffer [250 mM HEPES‐NaOH (pH 7.7), 1 mM EDTA and 0.1 mM neocuproine], supplemented with 100 μM deferoxamine. Homogenates were sonicated and centrifuged at 12,000 rpm (4°C) for 10‐15 min, treated with DTT, NaHS or GYY4137 for in vitro sulfhydration. After cell treatment with DTT, NaHS or GYY4137, or transfection with adenovirus, cell homogenates were collected for in vivo sulfhydration. Cell lysates were added blocking buffer (HEN buffer adjusted to 2.5% SDS and 20 mM MMTS) then incubated at 50℃ for 20 min with frequent vortex. The MMTS was then removed by acetone, and the proteins were precipitated at −20℃ for 20 min. Resuspended proteins in HENS buffer (HEN buffer adjusted to 1% SDS) and added in 4 mM biotin‐HPDP for 3 hours at 25℃, incubated overnight with magnetic beads. Then, biotinylated protein was pulled down by streptavidin magnet beads and eluted by SDS‐PAGE loading buffer for Western blot analysis.

### 2.10 PDI activity assay

PDI activity assay was performed as described by Appenzeller‐Herzog et al.^15^ The proteins without denatured were separated by using non‐reducing SDS‐PAGE on 12% (w/v) native‐polyacrylamide gels. To prevent protein denaturation, make sure the entire electrophoresis and transfer processes are performed on ice, and do not add any denaturants and reducing agents throughout the process. The other steps are same as Western blot. The bands of oxidative‐PDI (ox‐PDI, active protein) and reduction‐PDI (red‐PDI, inactive protein) appear at 60 KD and 150 KD, respectively. The ratio of ox‐PDI/red‐PDI acts as PDI activity.

### 2.11 Statistical analysis

Data are presented as means ± SD. Differences among groups were analysed by one‐way ANOVA, then Student‐Newman‐Keuls test. *P* < 0.05 was considered statistically significant.

## 3 RESULTS

### 3.1 H_2_S donor's treatment reduced HHcy‐promoted plaque size and ER stress

**FIGURE 1:**
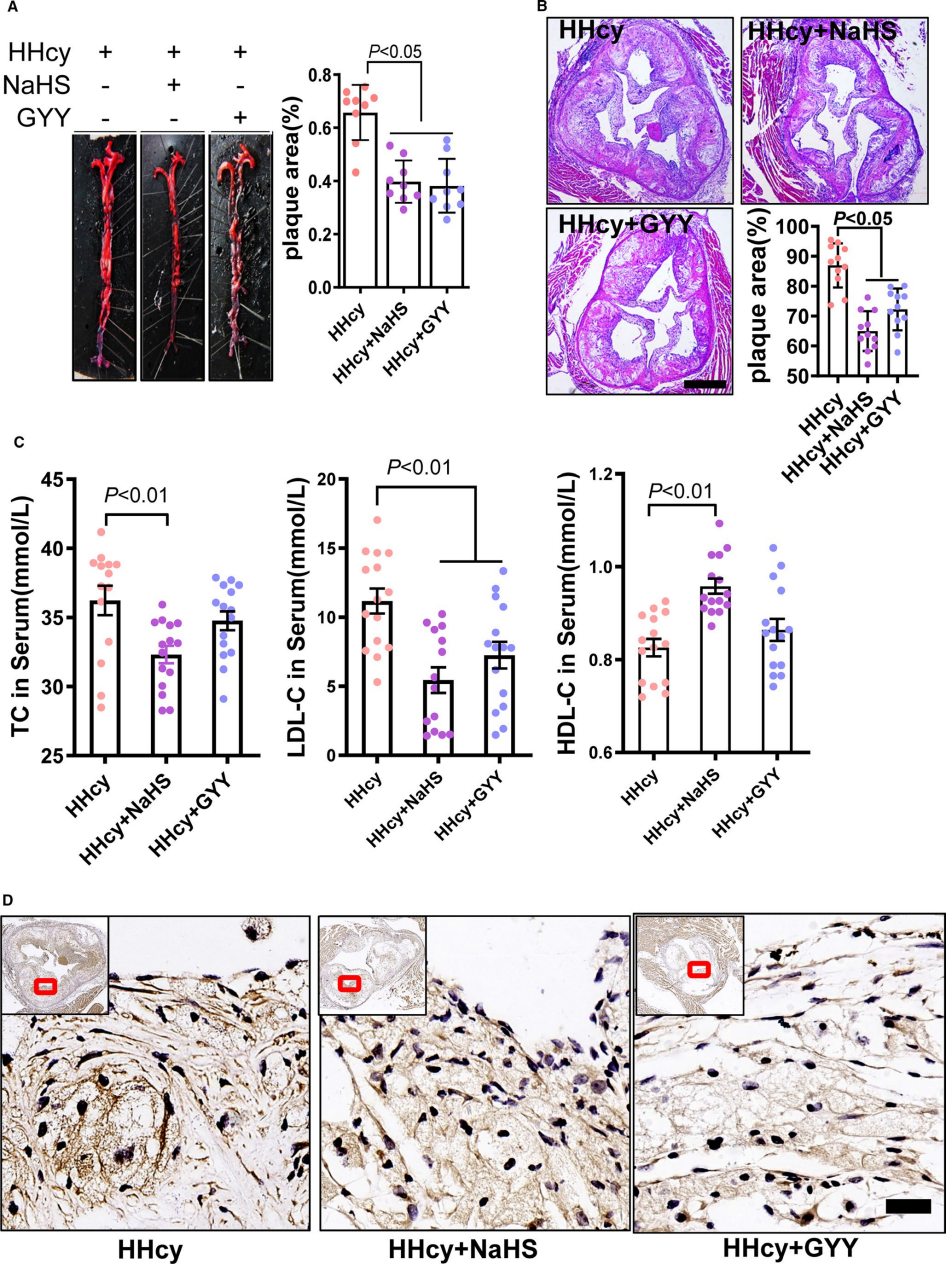
H_2_S donor treatment lowered plaque area and ER stress in atherosclerosis with HHcy mice. A, Aorta plaque area was measured by en‐face Oil‐red O staining. B, Aortic root plaque size was detected by H&E staining (bar = 500 μm). C, Serum total cholesterol, LDL‐cholesterol (LDL‐c), HDL‐cholesterol (HDL‐c) changes after H2S donor treatment. D, ER stress marker protein‐GRP78 immunohistochemical staining was assayed in aortic root plaque (bar = 20 μm). Small images in the left corner are overall perspective pictures

To investigate the effects of H_2_S on atherosclerotic development and ER stress of plaque with HHcy, we generated a mouse model by feeding ApoE knockout mice with Paigen diet and L‐methionine in drinking water (1 g/kg) for 12 weeks as our previous study.^10^ H_2_S donors NaHS and GYY4137 were administered by intraperitoneal injection (twice/day), with normal saline as a control. By aortic en‐face Oil Red O staining, the atherosclerotic plaque areas were decreased with H_2_S donor (NaHS and GYY4137) treatment (Figure 1A). Consistently, aortic root plaque size was also lowered using haematein and eosin staining (Figure 1B). Here, we also measured the serum lipid profile and found that NaHS lowered serum total cholesterol (TC) and LDL‐cholesterol (LDL‐c) but slightly increased HDL‐cholesterol (HDL‐c) (Figure 1C). By immunohistochemical staining for ER stress marker GRP78, we confirmed that GRP78 dominantly expressed in lumen layer of the plaque, and which significantly reduced by H_2_S donors (Figure 1D). These data indicated that H_2_S attenuated the atherosclerotic plaque development and ER stress response by HHcy.

### 3.2 H_2_S donor inhibited Hcy‐induced PDI dysfunction and ER stress

**FIGURE 2:**
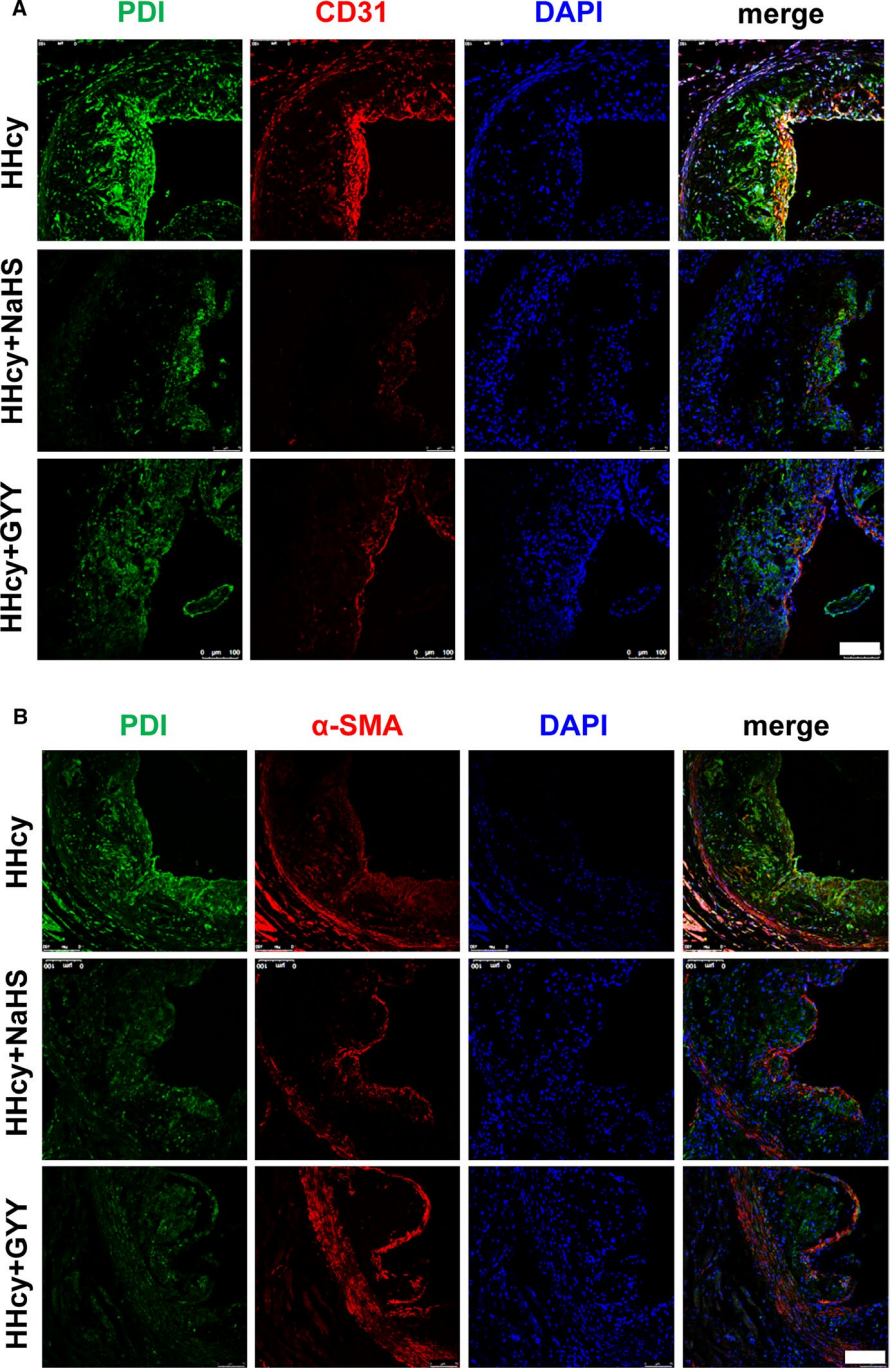
H_2_S donor reduced PDI protein expression in atherosclerotic plaques. A, Immunofluorescence staining showed the PDI (green) expression in CD31 (red)‐positive cells of plaque. Bar = 100 μm. B, PDI expression in α‐SMA (red)‐positive cells of plaque. Bar = 100 μm

PDI is wildly expressed in various cells. We first identified the PDI expression in the endothelial cells and vascular smooth muscle cells (VSMCs) within the plaque. In the plaque of atherosclerosis with HHcy, PDI majorly co‐localized in the CD31‐positive cells (endothelia derived; Figure 2A), and partly expressed in α‐SMA positive cells (VSMC derived, Figure 2B). Both NaHS and GYY4137 treatments reduced the PDI co‐localization in the CD31‐positive cells and α‐SMA–positive cells in the plaque, especially (Figure 2A and B) association with GRP78 attenuation (Figure 1D).

**FIGURE 3:**
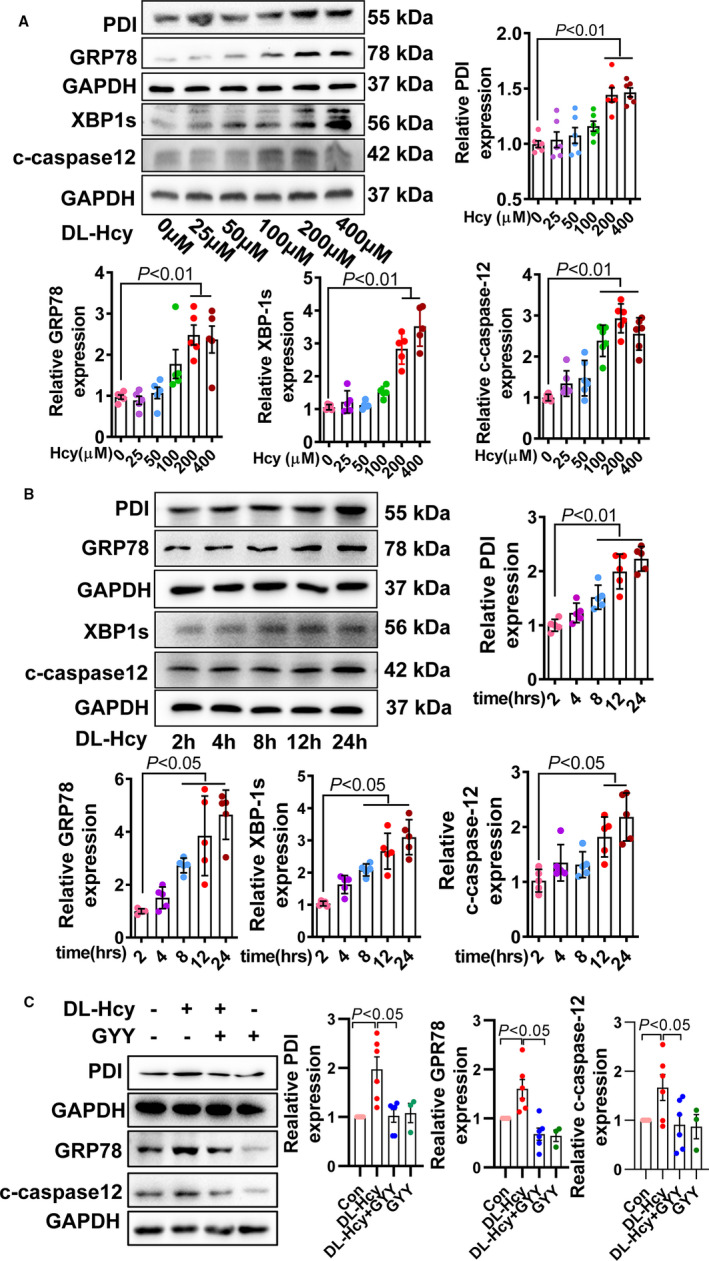
H_2_S donor lowered Hcy‐induced ER stress in human aortic endothelial cell (HAEC). A, ER stress markers including early markers PDI and GRP78, late markers spliced XBP1 (XBP1s) and cleaved‐caspase 12 (c‐caspase 12) were assayed by Western blot after different concentration of DL‐Hcy (from 25 to 400 μM) treatment for 24 h. B, The ER stress markers change while DL‐Hcy (200 μM) treated HAEC for different times (from 2 to 24 h). C, H_2_S donor‐GYY4137 reduced ER stress markers induced by Hcy (200 μM for 24 h)

To confirm these effects of H_2_S, we cultured the human aortic endothelial cell (HAEC) as DL‐Hcy as stimulator. DL‐Hcy dose dependently (from 25 to 400 μM; Figure 3A) and time dependently (from 2 to 24 hours; Figure 3B) increased PDI protein expression, consistent with ER stress activation‐GRP78, spliced x‐box‐binding protein 1 (XBP1s), cleaved‐caspase‐12 (c‐caspase 12) expression. According to these data, we selected DL‐Hcy (200 μM) treatment HAEC for 24 h in following experiments. In this cellular model, we confirmed that H_2_S donor lowered Hcy‐induced PDI elevation and ER stress (Figure 3C).

**FIGURE 4:**
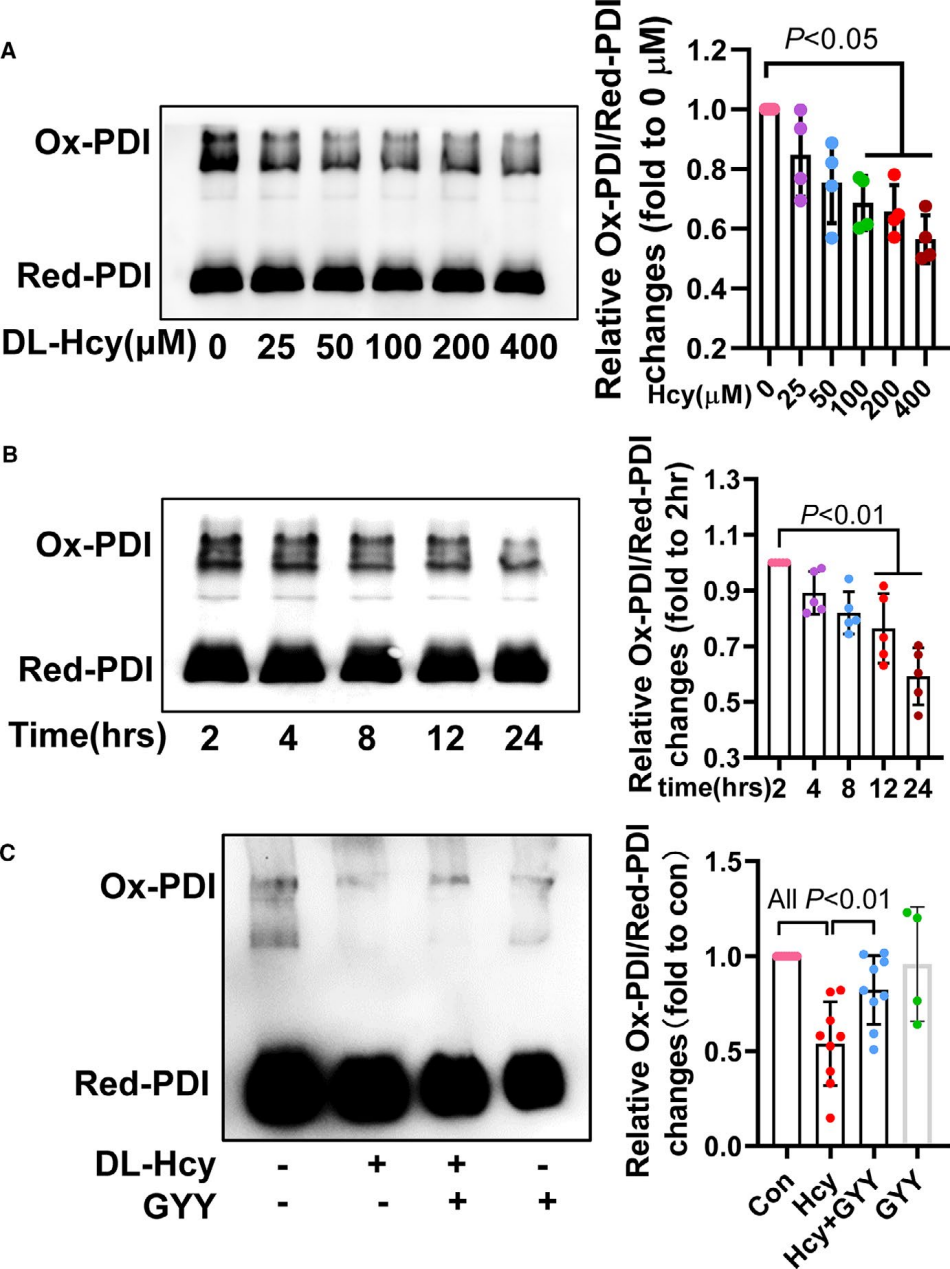
H_2_S rescued HHcy‐induced PDI activity inhibition. A, PDI activity assessment with oxidative‐PDI/reduction‐PDI by native‐polyacrylamide gel electrophoresis, after different concentration of DL‐Hcy treatment for 24 h. B, PDI activity changes after treating HAEC with DL‐Hcy for different times. C, GYY4137 action on HHcy‐induced PDI activity inhibition

PDI activity‐specific oxidoreductase activity tightly linked with misfold protein causing unfold protein response (UPR) and apoptosis.^13^ Oxidized PDI is activated form of PDI, so we detected ratio of oxidized PDI/reduced PDI as PDI activity. Here, we found that DL‐Hcy also reduced PDI activity in dose‐dependent (Figure 4A) and time‐dependent (Figure 4B) manner. The inhibition of PDI activity was reversed by H_2_S donor (Figure 4C). These data highlight HHcy increased UPR, in part due to the PDI dysfunction, and which were reversed by H_2_S treatment.

### 3.3 H_2_S sulfhydrated PDI to promote PDI activity

**FIGURE 5:**
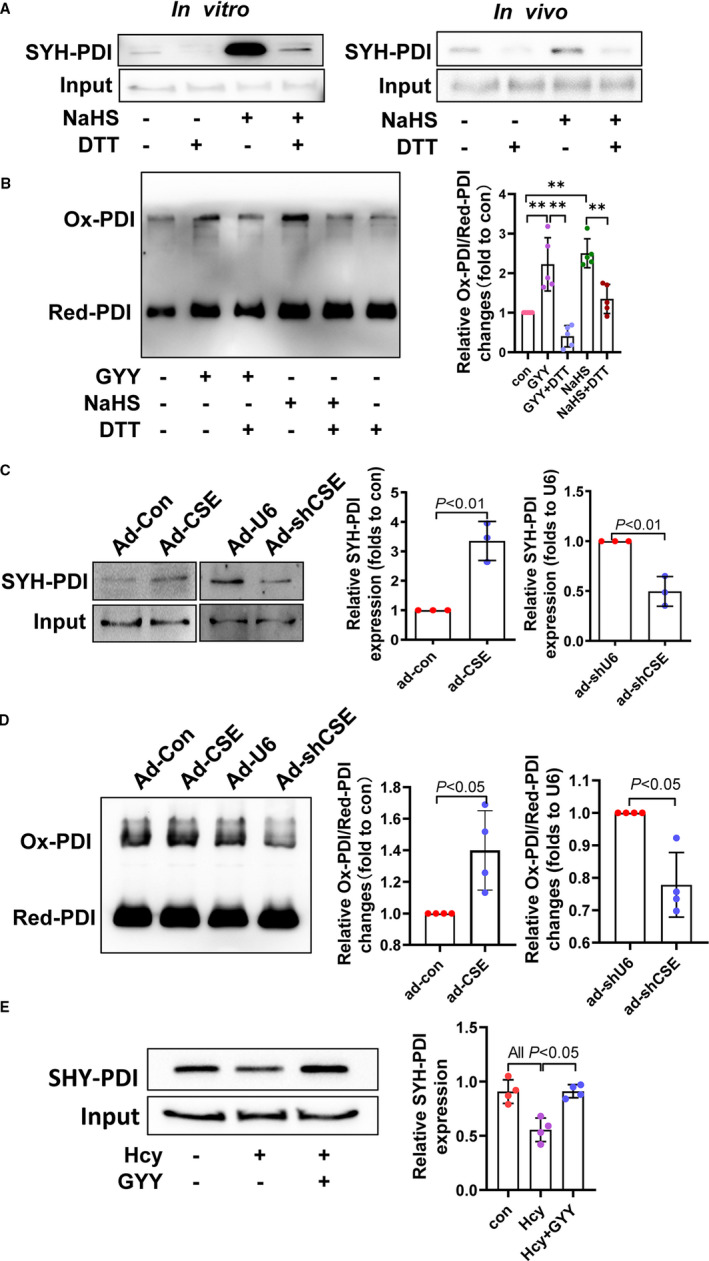
Endogenous CSE/H_2_S system in PDI sulfhydration and PDI activity. A, In vivo and In vitro PDI sulfhydration were identified by biotin switch assay. B, PDI sulfhydration associated with PDI activity. C, PDI sulfhydration changes while overexpressed cystathionine γ lyase (CSE) or knockdown CSE by adenovirus. D, Overexpression or knockdown CSE‐associated PDI activity changes. E, HHcy and H2S donor (GYY4137) treatment‐induced PDI sulfhydration changes in HAECs

Protein sulfhydration at the cysteine residue is a novel translational modification attribution to protein functional regulation.^14^ To investigate whether the PDI activity is dependent on its sulfhydration, we detect it by biotin switch assay. In vitro, cellular lysis incubated with NaHS, and the PDI sulfhydration was confirmed and which could be removed by DTT (sulfhydration remover, 100 mM). In cultured HAEC, NaHS (1 mM) or DTT (1 mM) treatment for 2 hours, endogenous PDI sulfhydration was also verified (Figure 5A). In keeping with PDI sulfhydration, PDI activity also elevated by NaHS and GYY but reduced by DTT (Figure 5B).

Since endogenous H_2_S generation in endothelium is majorly dependent on CSE, we found that overexpression or knockdown CSE using adenovirus enhanced or reduced PDI sulfhydration (Figure 5C). Accordingly, PDI activity also increased by overexpression of CSE or decreased by knockdown CSE (Figure 5D). Corresponding to PDI activity by Hcy, DL‐Hcy also down‐regulated endogenous PDI sulfhydration, which was reversed by GYY4137 (Figure 5E). All of data indicate that sulfhydration is a novel functional post‐translational modification of PDI by CSE/H_2_S, modulation its activity.

### 3.4 PDI sulfhydration at C53/57 and C397/400 sites

**FIGURE 6:**
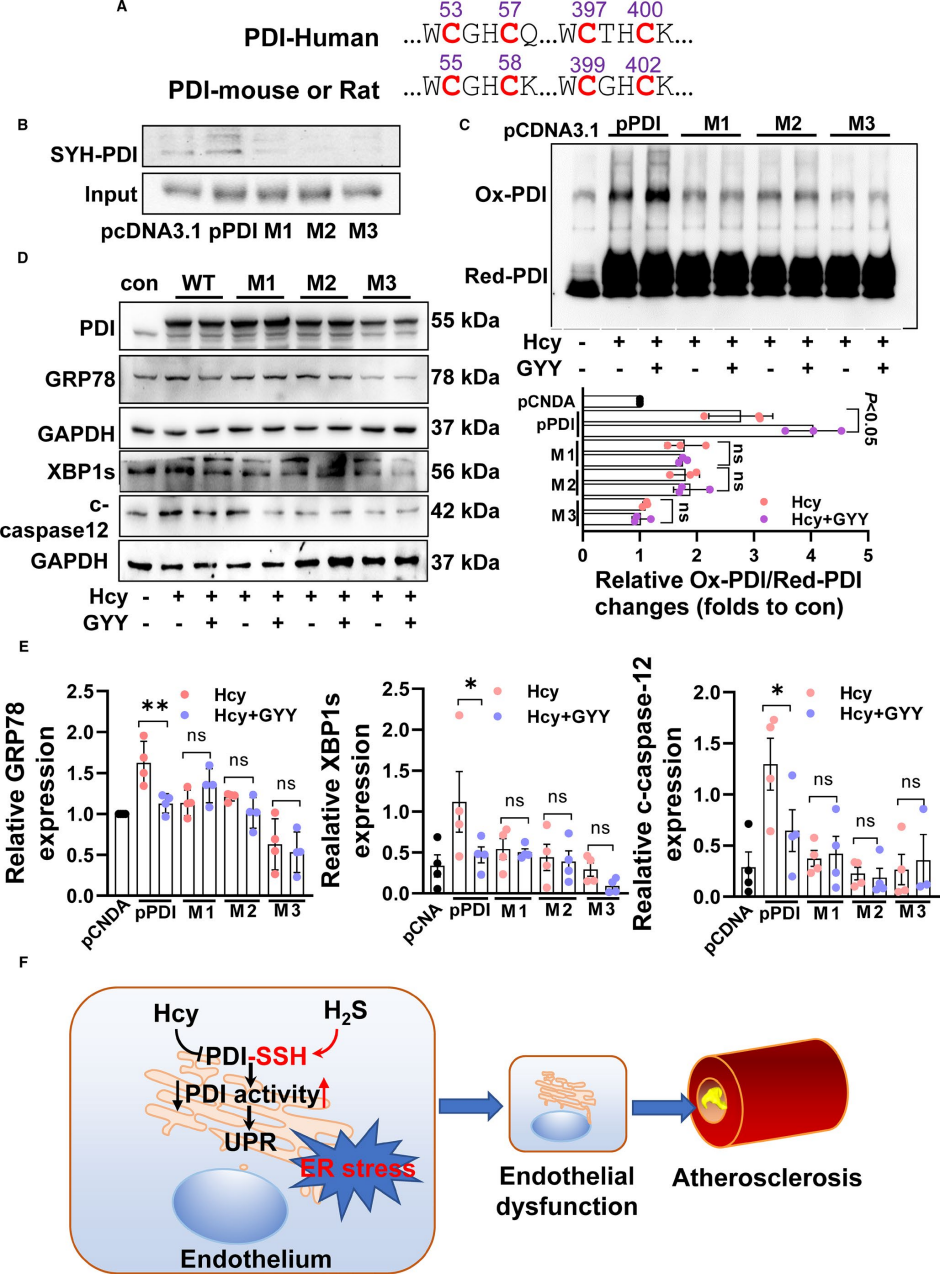
PDI sulfhydration sites and their activity. A, Two conserved cysteine‐terminal CXXC in humans, rat and mouse. B, H_2_S induced PDI sulfhydration changes by transfection wild‐type human PDI (pPDI) plasmid, mutation C53/57 (M1) plasmid, mutation 397/400 plasmid (M2) and mutation four cysteine site (M3) plasmid into HEK‐293 cells. C, H_2_S‐promoted PDI activity changes while mutation sulfhydration sites of PDI. D, H_2_S reduced HHcy‐induced ER stress while mutation sulfhydration sites of PDI. E, The relative protein expression of GRP78, XBP1s and cleaved‐caspase‐12. **P < 0.01, *P < 0.05. F, Schematic diagram of the present findings

PDI protein has conservative two CXXC domains (Figure 6A) which might be potential sulfhydration sites. To determine the sulfhydration sites, we constructed PDI mutation plasmids: M1 (C53S + C57S), M2(C397S + C400S) and M3(C53S + C57S+ C397S + C400S) as pcDNA3.1 as blank control and then transfected them into HEK‐293 cells. After transfection for 24 hours, giving GYY4137 (1 mM) incubation for 2 hours, then the cells were collected and PDI sulfhydration was measured. As Figure 6B showed, all M1, M2 and M3 blocked PDI sulfhydration, indicating two CXXC domains could be sulfhydrated by H_2_S. In line with the sulfhydration blocking, H_2_S‐promoted PDI activity was also blocked by these mutations (Figure 6C). To verify the PDI sulfhydration association with ER stress, we treated these HEK‐293 cells with DL‐Hcy and GYY4137 for 24h. In wild‐type PDI, GYY4137 lowered Hcy‐induced GRP78, XBP1s and cleaved‐caspase‐12 expression, which was blocked by M1, M2 and M3 mutation (Figure 6D). These data suggest that PDI sulfhydration at C53/57 and C397/400 sites partly contributes to reducing HHcy‐induced ER stress effect of H_2_S.

## 4 DISCUSSION

In the present study, we first demonstrate that CSE/H_2_S sulfhydrates PDI at C53/57, C397/400 sites facilitating its protein folding activity, then reducing unfold protein response (final process of ER stress) by HHcy, attenuating endothelial dysfunction and atherosclerosis development with HHcy (Figure 6E). This study also highlights a novel molecular mechanism of H_2_S on anti‐cardiovascular injuries of HHcy, except for scavenging reactive oxygen species^11^ and lowering Hcy by sulfhydrating CSE.^10^

Hyperhomocysteinaemia is an independent risk factor of cardiovascular disease, especial atherosclerosis.^1^ Although B type vitamins or folic acid interventions can lower total Hcy, these therapies did not effectively prevent cardiovascular events by serial meta‐analyses,^16^, ^17^, ^18^, ^19^ in part due to these intervention failures to repair the existing damage by HHcy. H_2_S is a novel protective gasotransmitter for atherosclerosis.^20^ H_2_S could reduce HHcy‐induced cardiac, aortic, VSMC and endothelial oxidative stress injuries^11^, ^21^, ^22^, ^23^; inhibit platelet activation^24^; anti‐apoptosis–derived mitochondrial dysfunction^25^; decrease lipid peroxidation^26^ and attenuate vascular permeability,^27^ all of which contribute to pathogenesis of atherosclerosis. Here, we demonstrated that H_2_S prevented HHcy‐induced endothelial ER stress to reduce endothelial dysfunction, thus attenuating the atherosclerosis development.

Elevated Hcy caused endothelial dysfunction including ER stress‐induced apoptosis, similar to other ER stress inducers tunicamycin and thapsigargin.^3^ However, the underlying mechanism of HHcy‐inducing ER stress is almost unknown. PDI is a prototypic thiol isomerase that catalyses the formation and cleavage of thiol‐disulphide bone.^28^ PDI also functions as a molecular chaperone that prevents misfolded protein aggregation.^29^ About 30% proteins required PDI to form disulphide bone. Oxidized PDI binding to unfold protein‐free sulfhydryl facilitates protein folding with endoplasmic reticulum oxidoreductin 1.^12^ So, the oxidized PDI is activated form of PDI. Ox‐LDL inhibited PDI causing ER stress and endothelial apoptosis to promote atherosclerosis.^13^ Here, we found that HHcy dramatically elevated PDI protein expression in endothelial‐derived cells of plaque, association with ER stress. In HAECs, HHcy also up‐regulated PDI and ER stress markers, but down‐regulated PDI activity. These data highlight that PDI activity inhibition is the molecular mechanism of HHcy‐inducing endothelial UPR. Furthermore, we found that H_2_S donor significantly enhanced PDI activity per se, and reversed the HHcy effects on PDI expression and activity. This finding also indicates that PDI activity is a regulatory target of H_2_S.

PDI activity tightly associated with endothelial function. PDI associates with mitochondrial fission protein‐Drp1 to reduce its redox status and activity, causing modulating endothelial senescence.^30^ Nox4 association with PDI regulated eNOS uncoupling by HSP90 to inhibit nitric oxide generation and endothelia‐dependent dilation.^31^ Extracellular PDI interacted with nitric oxide or vitronectin to regulate thrombus formation.^32^, ^33^ PDI also regulated von Willebrand factor dimerization to participate coagulation.^34^ NADPH oxidase‐derived reactive oxygen species (ROS) mediated cytoplasm PDI activity, and ROS‐mediated specific oxidase such as Ero1, Prx4, GPx7/8 and VKOR regulate PDI activity in ER.^35^ CSE/H_2_S has anti‐oxidative stress effect and directly scavenge ROS including peroxide and superoxide induced by HHcy.^11^ Thus, H_2_S supplemental attenuated ROS and oxidative‐reduction environment of ER, enhancing the PDI activity to correct the misfold protein and UPR.

Post‐translational modification of PDI is also associated with PDI activity. Phosphorylation at serine 357 site of PDI induces an open confirmation of PDI and turns it from a ‘foldase’ into ‘holdase’, mediates ER stress.^36^ Glutathionylation at two CXXC domains of PDI inhibited its isomerase activity trigging UPR.^37^ PDI is also nitrosylated at CXXC domains, leading to accumulation of polyubiquitinated proteins and activation of UPR.^38^ Here, we identified a new post‐translational modification‐sulfhydration of PDI at the same sites of nitrosylation or glutathionylation. Sufhydrated PDI inhibited ER stress induced by HHcy, preventing endothelial dysfunction.

Collectively, our present study highlights a new molecular mechanism of H_2_S preventing endothelial function under HHcy, attenuating atherosclerosis. We also demonstrated a new post‐translational modification, sulfhydration of PDI, which activate PDI to reduce ER stress. Some H_2_S‐releasing donors such as SG1002 for heart failure are in phase I clinical trials^39^; ATB‐346 for anti‐inflammation in a phase II clinical trial ^40^ showed some beneficial effects. Our work offers a further possibility of these drugs for atherosclerosis with HHcy therapy.

## CONFLICT OF INTEREST

The authors confirm that there are no conflicts of interest.

## AUTHOR CONTRIBUTIONS

Shan Jiang: conceptualization (equal); Data curation (equal); Formal analysis (equal); Investigation (equal); Methodology (equal); Writing‐original draft (equal). Wenjing Xu: data curation (equal); Formal analysis (equal); Investigation (equal); Writing‐original draft (equal). Zhenzhen Chen: funding acquisition (equal); Methodology (equal); Validation (equal). Changting Cui: data curation (equal); Formal analysis (equal); Methodology (equal); Validation (equal). Xiaofang Fan: methodology (equal); Resources (equal); Supervision (equal). Jun Cai: funding acquisition (equal); Investigation (equal); Resources (equal); Supervision (equal). Yongsheng Gong: project administration (equal); Supervision (equal); Writing‐original draft (equal); Writing‐review & editing (equal). Bin Geng: formal analysis (equal); Funding acquisition (equal); Project administration (equal); Validation (equal); Writing‐original draft (equal); Writing‐review & editing (equal).
